# Multimodal prediction of pain and functional outcomes 6 months following total knee replacement: a prospective cohort study

**DOI:** 10.1186/s12891-022-05239-3

**Published:** 2022-03-29

**Authors:** Robert R. Edwards, Claudia Campbell, Kristin L. Schreiber, Samantha Meints, Asimina Lazaridou, Marc O. Martel, Marise Cornelius, Xinling Xu, Robert N. Jamison, Jeffrey N. Katz, Junie Carriere, Harpal P. Khanuja, Robert S. Sterling, Michael T. Smith, Jennifer A. Haythornthwaite

**Affiliations:** 1 Department of Anesthesiology, Perioperative & Pain Medicine, Harvard Medical School, Brigham & Women’s Hospital, Pain Management Center, 850 Boylston St, MA 02467 Chestnut Hill, USA; 2 Department of Psychiatry & Behavioral Sciences, Johns Hopkins University School of Medicine, Baltimore, Maryland USA; 3 Faculties of Dentistry & Medicine, McGill University, Strathcona Anatomy & Dentistry building 3640 University Street, Montreal, Qc H3A 2B2 Canada; 4 Departments of Medicine and Orthopedic Surgery, Orthopedic and Arthritis Center for Outcomes Research, Harvard Medical School, Brigham & Women’s Hospital, Boston, MA USA; 5 McGill University, Montreal, Qc H3A 2B2 Canada; 6 Department of Orthopaedic Surgery, Johns Hopkins University School of Medicine, Baltimore, Maryland USA

**Keywords:** Post-Operative Pain, Knee, Arthroplasty, TKA, TKR, Negative Affect, Catastrophizing, Quantitative Sensory Testing, Sleep, Temporal Summation

## Abstract

**Background:**

Knee osteoarthritis (OA) is among the most common and disabling persistent pain conditions, with increasing prevalence and impact around the globe. In the U.S., the rising prevalence of knee OA has been paralleled by an increase in annual rates of total knee arthroplasty (TKA), a surgical treatment option for late-stage knee OA. While TKA outcomes are generally good, post-operative trajectories of pain and functional status vary substantially; a significant minority of patients report ongoing pain and impaired function following TKA. A number of studies have identified sets of biopsychosocial risk factors for poor post-TKA outcomes (e.g., comorbidities, negative affect, sensory sensitivity), but few prospective studies have systematically evaluated the unique and combined influence of a broad array of factors.

**Methods:**

This multi-site longitudinal cohort study investigated predictors of 6-month pain and functional outcomes following TKA. A wide spectrum of relevant biopsychosocial predictors was assessed preoperatively by medical history, patient-reported questionnaire, functional testing, and quantitative sensory testing in 248 patients undergoing TKA, and subsequently examined for their predictive capacity.

**Results:**

The majority of patients had mild or no pain at 6 months, and minimal pain-related impairment, but approximately 30% reported pain intensity ratings of 3/10 or higher. Reporting greater pain severity and dysfunction at 6 months post-TKA was predicted by higher preoperative levels of negative affect, prior pain history, opioid use, and disrupted sleep. Interestingly, lower levels of resilience-related “positive” psychosocial characteristics (i.e., lower agreeableness, lower social support) were among the strongest, most consistent predictors of poor outcomes in multivariable linear regression models. Maladaptive profiles of pain modulation (e.g., elevated temporal summation of pain), while not robust unique predictors, interacted with psychosocial risk factors such that the TKA patients with the most pain and dysfunction exhibited lower resilience and enhanced temporal summation of pain.

**Conclusions:**

This study underscores the importance of considering psychosocial (particularly positively-oriented resilience variables) and sensory profiles, as well as their interaction, in understanding post-surgical pain trajectories.

## Background

Knee osteoarthritis (OA) is one of the leading causes of pain and disability in the United States and across the globe, accounting for millions of years lived with disability, substantially reduced quality of life, and hundreds of billions of dollars in direct and indirect costs every year in the U.S. alone [1–5]. Prominent symptoms of knee OA include decreased range of motion, stiffness, limitations in physical mobility, and function-limiting pain [6–9]. As with many chronic pain conditions, the mechanisms of pain in knee OA are complex, multifactorial, and incompletely understood [10, 11]. Numerous studies have documented that radiographic markers of disease severity are relatively weak correlates of pain severity and disability [12–16], suggesting that an array of other biopsychosocial mechanisms may more meaningfully contribute to variability in the experience of joint pain in patients with OA [17–21].

Early OA treatment generally focuses on symptom management and interventions aimed at slowing the rate of disease progression. However, as no structure-modifying medications have been approved, total joint replacement is, for many patients in the later stages of the disease, an effective intervention to reduce pain and improve physical functioning [7, 22–26]. Primary total knee arthroplasty (TKA) is one of the most common orthopedic surgeries, with over 700,000 procedures performed annually in the U.S. and substantial projected increases in the coming years [27, 28]. Recent studies have brought a growing recognition of the tremendous inter-patient variability in pain-related outcomes after surgery [29–33]. Following nearly any operative procedure, a substantial percentage of patients reports persistent post-operative pain [31, 34, 35]. TKA outcomes similarly show great variability, with some patients reporting full resolution of knee pain, and large improvements in physical capacity, while others report continuing, or even worsening, pain. Recent reviews suggest that approximately 25–30% of TKA patients do not achieve satisfactory outcomes following surgery, prompting interest in identifying factors that are associated with either positive or negative outcomes [2, 36–40]. Indeed, it is clear that many patients persistently complain of significant knee pain following TKA despite normal radiographs, unremarkable physical examinations, and even self-reported “good” results [41–47]. Moreover, individuals who have undergone TKA remain significantly lower than age-related population norms on measures of health-related quality of life [4, 48–53].

With the rapid growth of TKA rates and the recognition that a sizable minority of patients obtain little benefit from the surgery, attention has turned to the identification of risk factors for inadequate improvement in pain and function after joint replacement. Psychosocial processes have shown consistent influence in shaping the long-term course of post-TKA outcomes [44, 54–58], with anxiety and catastrophizing being prominent risk factors [59, 60], but research in this area (e.g., biopsychosocial predictors of post-TKA outcomes) remains at a relatively early stage. Some high-quality longitudinal studies [61] and reviews [62] find only modest support for a predictive association. Collectively, though, recent systematic reviews have tended to support the importance of negative affective and cognitive processes [28, 36, 63]. For example, Sorel and colleagues reported poorer outcomes in patients who preoperatively had elevated scores on measures of pain catastrophizing, increased symptoms of anxiety and depression, and somatization [63]. Impaired sleep, or insomnia, has also emerged as an important predictor of deleterious pain-related outcomes after surgery [64–66]. However, one aspect of psychosocial contributors to TKA outcomes that has clearly been under-studied is the impact of positive, or resilience-related, psychosocial characteristics [67–69].

Sensory profiling of OA patients using quantitative sensory testing (QST) in order to measure and quantify sensitization-related processes has become an increasingly common assessment method in patients with chronic musculoskeletal pain [14, 70–77]. As recent reviews note, widespread hyperalgesia, elevated temporal summation of pain, and deficits in endogenous pain inhibition are present in a substantial proportion of knee OA patients [70, 72, 74], and have the potential to influence long-term pain outcomes, including trajectories after joint replacement [72, 78, 79]. Findings from prospective TKA studies are mixed [80], but some findings suggest that indices of sensitization (e.g., high levels of temporal summation of pain, low pain thresholds), assessed pre-surgically, are associated with worse short- and long-term outcomes following TKA, such as: elevated pain severity, increased opioid use, lower patient satisfaction, and reduced physical function [60, 81–87].

To date, no multi-site studies have comprehensively examined psychosocial, clinical, sensory, and functional phenotypic factors as predictors of pain and functional outcomes after TKA. The present investigation sought to characterize, with detailed pre-operative phenotyping, multiple predictors of pain and physical function 6 months after total knee replacement. The objective of the study was to identify unique predictor domains, recognizing that each distinct domain may contain multiple inter-related variables, as well as to explore potential interactions between psychosocial and sensory profiles in patients undergoing unilateral TKA. We hypothesized that higher levels of negative affect, lower levels of resilience-related factors, and elevated indices of hyperalgesia and pain facilitation would be associated with greater report of knee pain and pain-related functional impact at 6 months post-surgery.

## Methods

### Study population

Subjects in this prospective, longitudinal observational study met the American College of Rheumatology criteria for knee OA and were scheduled to undergo unilateral primary TKA at either Brigham & Women’s Hospital (Boston, MA) or Johns Hopkins Bayview Medical Center (Baltimore, MD). These academic teaching hospitals serve large urban and suburban catchment areas surrounding the Boston and Baltimore metro regions, respectively. Additional study inclusion criteria included: age of 45 years or greater and adequate fluency in English to complete self-report questionnaires. Exclusion criteria included: disorders of cognition preventing completion of the study procedures, recent history of a myocardial infarction, presence of an autoimmune disorder, severe Raynaud’s symptoms, and documented peripheral neuropathy of at least moderate severity. Potential participants were identified by posted advertisements. The institutional review boards of both Brigham and Women’s Hospital and Johns Hopkins University approved all study procedures, and written informed consent was obtained from all participants. The study is registered as NCT01370421. All study methods were performed in accordance with the Declaration of Helsinki.

### Data collection

A comprehensive phenotyping visit was completed by patients in the pre-operative period, approximately 2 weeks before surgery. This visit included a medical history and clinical assessment, psychosocial evaluation, quantitative sensory testing, and physical function testing. The pre-specified primary outcome measures (covering domains of general and knee-specific pain and function) for the study, which were assessed pre-operatively and at 6 months following surgery, were: (1) The Brief Pain Inventory (BPI) Pain Severity subscale, which assesses pain intensity on a 0–10 NRS; (2) the BPI Pain Interference subscale, which measures the impact of pain on a number of functional activities (e.g., work, recreation, socializing) [88, 89]; (3) The Western Ontario McMaster Universities Scales (WOMAC) pain scale; and (4) the WOMAC function scale [90, 91]. WOMAC items were scored on a 0–100 visual analog scale and averaged to compute pain and function scale scores [92, 93]. Surgical data (e.g., duration of surgery, type of anesthesia) were collected from the electronic medical record following the procedure. Study participants received inpatient physical therapy following the surgery, and then were referred for outpatient rehabilitation services (e.g., physical and occupational therapy) following discharge.

### Questionnaires

Patients self-reported demographic information, including age, sex, race, marital status, education, and employment on a standard patient history questionnaire. Clinical measures included body mass index (BMI), patient-reported presence of additional (non-OA) chronic pain, patient-reported neuropathic pain (yes/no), prior surgery in the index knee, smoking status (including number of cigarettes per day), number of alcohol-containing drinks per week, and use of analgesic medications (including NSAIDs, antidepressants, and others). Additional self-report assessments of pain and health (in addition to the BPI and WOMAC, the primary outcome measures) consisted of the Widespread Pain Index (WPI) [94], the SF-36 General Health subscale [95], the EuroQOL [96], and the Godin exercise questionnaire [97]. We administered a number of questionnaires assessing psychosocial factors, which have frequently appeared as predictors of post-surgical pain-related outcomes. Questionnaires were chosen based on strong psychometric validation characteristics, as well as previous association with persistent pain. The Pain Catastrophizing Scale (PCS), which has been validated in pain patients and controls, was used to measure catastrophic thinking associated with pain [98]. Depressive symptoms and anxiety were assessed using short-form instruments from the NIH roadmap initiative, Patient Reported Outcome Measurement Information System (PROMIS), which have been extensively validated in studies comparing results with established scales, and have been calibrated on over 20,000 persons [99]. The Brief Symptom Index 18-Somatization Scale [100] was used to measure somatization. We administered the NEO to assess personality characteristics [101]. Positive, resilience-related measures included the positive affect subscale of the PANAS [102], 0–100 ratings of expectations for improvement following surgery, and the ENRICHD Social Support Instrument (ESSI) [103]. Multiple sleep-related assessments were included as well. Patients completed the Pittsburgh Sleep Quality Index (PSQI) [104], the Insomnia Severity Index (ISI) [105], and a 0–100 scale assessing fatigue severity [106].

### Physical function

Two physical function tests were administered: a stair-climbing task and a 6-min walk task which we have utilized in previous knee OA studies [53, 107]. Pain ratings (0–100) were recorded during both tasks, along with functional measures such as the distance walked in 6 min and the time taken to ascend and descend a flight of 10 stairs. In addition, participants completed situational pain catastrophizing scales (SPCS) following each task to assess the degree of catastrophizing experienced during the functional testing.

### Quantitative sensory testing

#### Mechanical pain

As in our prior OA work [14, 82], mechanical pain sensitivity was evaluated using a handheld algometer, pinprick stimuli, and cuff algometry. A digital pressure algometer (Somedic) was used to assess mechanical pain thresholds. Pressure pain thresholds (PPThs) were determined bilaterally at the trapezius muscle and the patella. At each site, mechanical force was applied using a 0.5 cm2 probe covered with polypropylene pressure-transducing material; pressure was increased at a steady rate of 30 kPA/s until the subject indicated that the pressure was "first perceived as painful".

Mechanical temporal summation was assessed with the use of weighted DFNS (German Research Network of Neuropathic Pain) probes, which are metal pinprick stimulators of various weights, at the patella and the middle phalange of the third digit on the non-dominant hand. The stimulus was first applied once for 1 s, using the 128 mN and 256 mN probes, and pain ratings were noted. After that, both probes were used to apply a series of 10 consecutive 1-s stimuli, and pain ratings were obtained for the stimulus train. Temporal summation was operationalized as the difference between ratings of the 10-stimulus train and ratings of a single stimulus for the 256 mN probe.

Response to deep pressure pain was ascertained via cuff pressure algometry (CPA) using a Hokanson rapid cuff inflator. In brief, tonic, deep-tissue, mechanical stimulation was applied using a pneumatic tourniquet cuff, which was inflated gradually to determine the cuff pain threshold. A standard blood pressure cuff was placed around the subjects’ gastrocnemius muscle and was then inflated to an initial pressure of 60 mmHg. The pressure was steadily increased, at approximately 20 mmHg/s, until participants reached a “moderate” pain intensity rating of 4 out of 10, similar to our prior studies [108].

#### Cold pain sensitivity

Responses to noxious cold were evaluated using a cold pressor task (CPT), involving immersion of the dominant hand in a circulating cold water bath (NesLab RTE-17) maintained at 4 °C. Participants immersed their dominant hand (up to the wrist) in the water bath and maintained their right hand in the water bath until reaching pain tolerance (or a 3 min maximum). Participants rated the maximum intensity of the cold pain on a 0–10 scale (“no pain” to “most intense pain imaginable”) during and at the conclusion of the CPT. Painful aftersensations were assessed 30 s after the completion of the CPT.

#### Conditioned Pain Modulation (CPM)

CPM, a non-invasive test of endogenous pain-inhibitory systems using a heterotopic noxious conditioning paradigm, was assessed during two brief CPTs. During each, PPTh was assessed on the contralateral trapezius after 20 s of cold immersion. As in our prior work [71], we calculated a CPM Index that reflected the magnitude of change in PPTh during cold pressor relative to baseline. The CPM Index is calculated using the formula: (PPTh during the cold pressor test/baseline PPTh)*100. Scores over 100 indicate positive/effective CPM (i.e., pain threshold increased during the cold pressor test).

#### Self-report of pain sensitivity

Participant self-report of sensitivity to pain was assessed using the Pain Sensitivity Questionnaire (PSQ), a validated measure of perceived sensitivity to daily pain-producing events [109].

#### Actigraphy

Patients wore a Philips Respironics Actiwatch-2 continuously on the non-dominant wrist for 1 week, similar to previous studies [110]. The week of actigraphy assessment followed the pre-surgical assessment visit; data were averaged across days to calculate sleep continuity parameters: Sleep Efficiency, Total Sleep Time, and Wake After Sleep Onset Time according to standardized methods [111, 112].

## Statistical analysis

There were four outcomes measured pre- and post-surgery: BPI pain severity, BPI pain interference, knee pain on the WOMAC, and physical function on the WOMAC. Outcomes were measured prior to TKA and then at 6 weeks, 3 months, and 6 months after surgery. We pre-specified 6 months as the primary outcome time point, as this time point is frequently identified as clearly representing persistent post-operative pain [62]. The multisite nature of the study was intended to reduce bias and enhance generalizability. Total planned sample size for the study was 250 participants; study enrollment took place from 2012–2018. Changes in outcomes over time were examined using repeated measures ANOVAs. For each outcome, univariate association analyses were performed using Wilcoxon rank-sum tests, tests for Pearson correlation, and tests for Spearman correlations as appropriate. Variables with *p*-value < 0.1 were treated as potential predictors. After this initial testing, we carried out multiple imputation (with 20 imputations) in SAS software version 9.4 using *proc mi*. For highly inter-correlated predictors, we calculated the variance inflation factor (VIF), and variables with VIF > 2.5 were excluded, because the inclusion of them in the models might inflate variance, and thus affect the variable selection procedure. Therefore, the predictors that were included in the models initially were the ones that have univariate test *p*-value < 0.1 (in order to reduce the possibility of Type II error, as in prior predictive studies: [113]), as well as having VIF < 2.5. The same model was fitted in each imputed dataset, adjusting for study site, and results were pooled using proc mianalyze. Backwards variable selection was iteratively performed based on p-values. The above procedure was repeated for each outcome. The final models are shown in table form, along with univariate associations and descriptive data.

## Results

### Sample description

This multi-site sample of 248 subjects had a mean age of 65.1 ± 8.2. A majority (59.5%) of participants were women, and most (88%) reported their race as white. Well over half of the sample (62.4%) reported having at least a bachelor’s degree, and a plurality of participants (47.1%) were working either part- or full-time (see Table 1).

**Table 1:** Baseline demographic data

**Age**	65.1 ± 8.2
**Sex**	
% Female	59.5%
% Male	40.5%
**Race**	
% White	88.0%
% African-American	9.1%
% Other	2.9%
**Marital Status**	
% Married/Cohabiting	73.8%
% Single	8.1%
% Divorced/ Separated	11.1%
% Widowed	7.0%
**Education**	
% High School Only	12.0%
% Some College	25.6%
% College Graduate	29.3%
% Graduate Degree	33.1%
**Employment Status**	
% Paid work	47.1%
% Retired	39.3%
% Unemployed/Disabled	9.1%
% Other	4.5%

**Fig. 1:**
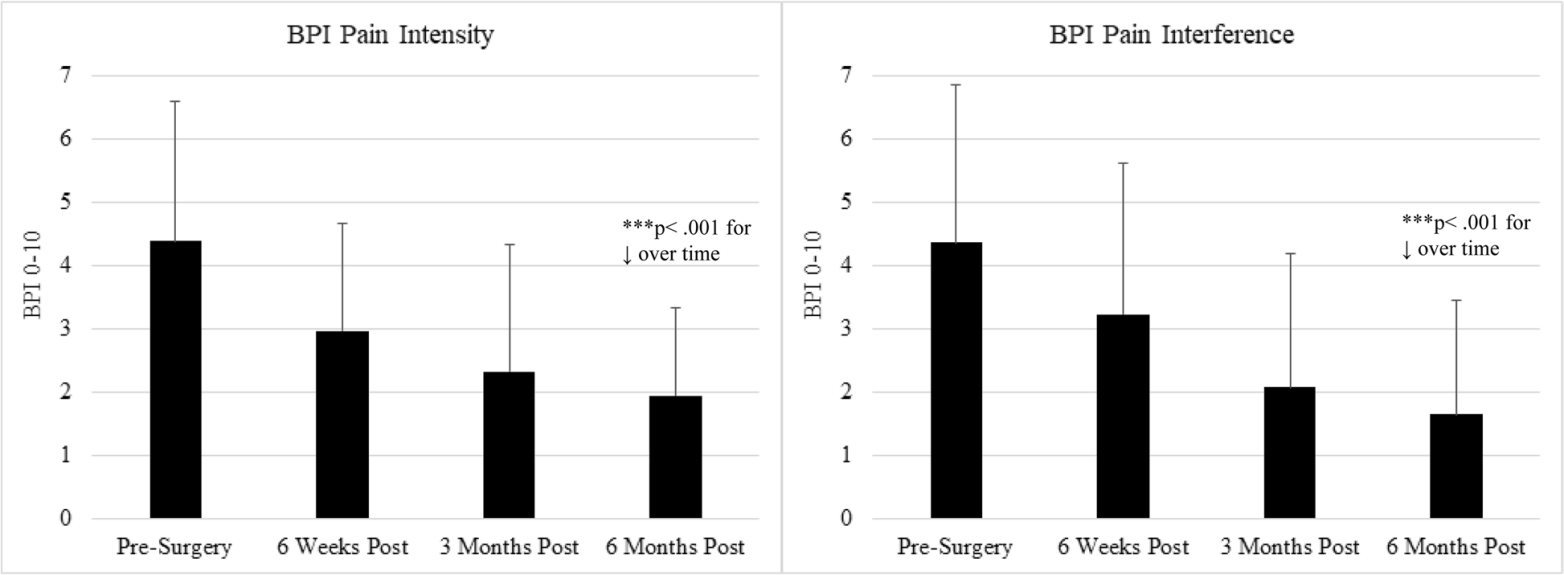
BPI Scores from pre-surgery to 6 months post-surgery (mean ± SD)

**Fig. 2:**
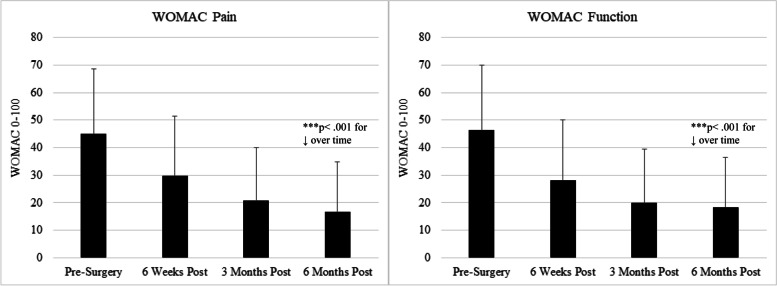
WOMAC Scores from pre-surgery to 6 months post-surgery (mean ± SD)

Pre-operatively, patients reported moderate pain and functional impairment, which improved steadily in the 6 months following surgery (see Figs. 1–2 and Table 2), with substantial variability across participants. Retention rates for follow-up assessments were as follows: 82.7% provided follow-up data at 6 weeks, 75.0% at 3 months, and 70.6% at 6 months.

**Table 2:** Categorical presentation of 6-month outcomes data

BPI Pain Severity at 6 months post-surgery	
Mean = 1.9 ± 1.8, Range = 0–10	
Range	% of Sample
0—0.99	27.0
1—1.99	29.4
2—2.99	13.5
3 – 3.99	16.0
4 – 4.99	6.1
5 +	8.0
BPI Pain Interference at 6 months post-surgery	
Mean = 1.6 ± 1.8, Range = 0–8.3	
Range	% of Sample
0—0.99	54.7
1—1.99	15.5
2—2.99	10.2
3 – 3.99	6.0
4 – 4.99	6.6
5 +	7.0
WOMAC Pain at 6 months post-surgery	
Mean = 17.5 ± 18.5, Range = 0–100	
Range	% of Sample
0—4.99	39.6
5—9.99	15.3
10—14.99	10.9
15—19.99	6.1
20—29.99	12.0
30—39.99	5.5
40 – 49.99	5.2
50 +	5.4
WOMAC Function at 6 months post-surgery	
Mean = 18.4 ± 18.1, Range = 0–97	
Range	% of Sample
0—4.99	28.9
5—9.99	21.9
10—14.99	13.3
15—19.99	6.6
20—29.99	13.3
30—39.99	4.3
40 – 49.99	4.3
50 +	8.4

The BMI for the sample was 31.1 ± 6.3, and 47.1% of the sample were ever smokers. On average, patients in the sample reported general health indicators consistent with typical scores for older adults in the U.S. [114]. See Table 3 for mean and variance values for potential predictors. Overall, 53.9% of the sample was having their right knee replaced, and 47.9% had a history of surgical procedures (e.g., arthroscopy) on the index knee, with a mean of 2 past knee surgeries for those with a surgical history. For medications, 31.4% were taking acetaminophen, 51.1% were taking NSAIDS or Cox-2 inhibitors, 15.8% reported taking antidepressants, and 9.5% reported using opioids.

**Table 3:** Univariate Associations with 6-Month outcomes (*p*-values)

Variable	Mean ± SD or %	BPI Severity	BPI Interference	WOMAC Pain	WOMAC Function
*Demographic Factors (97.1% complete data)*					
Age	65.1 ± 8.2	0.29	0.14	0.06	0.30
Sex	40.5% men	0.44	0.39	0.30	0.67
Race	88% white	0.41	0.16	0.54	0.12
Marital Status	70.8% married	0.47	0.54	0.83	0.68
Education	62.4% bachelor’s	0.07	0.15	0.001	0.001
Employment	47.1% working	0.79	0.78	0.70	0.90
*Clinical Factors (96.8% complete data)*					
BMI	31.1 ± 6.3	0.17	0.50	0.31	0.04
Presence of other (non-OA) chronic pain	23.7%	0.001	0.003	0.82	0.38
Number of painful body areas	2.6 ± 2.8	0.001	0.003	0.007	0.03
Patient-reported neuropathic pain	6.5%	0.08	0.04	0.81	0.83
Prior knee surgery	47.9%	0.87	0.30	0.44	0.66
Number of drinks per week	3.8 ± 4.9	0.56	0.27	0.30	0.08
Number of cigarettes per day	7.1 ± 12.5	0.81	0.13	0.27	0.30
Use of NSAIDS	51.1%	0.54	0.19	0.75	0.10
Use of acetaminophen	31.4%	0.80	0.71	0.18	0.78
Use of opioids	9.5%	0.02	0.03	0.01	0.009
Use of antidepressants	15.8%	0.71	0.43	0.15	0.37
*Surgical Factors (93.1% complete data)*					
Days in Hospital	2.7 ± 1.8	0.85	0.97	0.88	0.91
Surgical Duration (minutes)	107 ± 54	0.55	0.79	0.66	0.51
Type of Anesthesia	37.9% general	0.39	0.47	0.27	0.32
Femoral Nerve or Adductor Canal Block	35.1% yes	0.35	0.28	0.34	0.69
*Health and Function (96.4% complete questionnaire data, 76.2% complete function testing data)*					
SF-36 General Health	70.5 ± 17.1	0.99	0.23	0.003	0.009
EuroQOL	75.9 ± 16.6	0.10	0.05	0.02	0.05
Godin Exercise Questionnaire	26.8 ± 30.8	0.19	0.29	0.50	0.07
6-Minute walk distance (feet)	937.6 ± 291.7	0.38	0.13	0.12	0.03
Maximum pain during walking	41.6 ± 27.1	0.004	0.04	0.002	0.004
Stair-climbing time (seconds)	20.6 ± 9.4	0.85	0.58	0.86	0.51
Pain during stair climb	33.8 ± 25.3	0.03	0.28	0.001	0.004
*Psychosocial Factors (94.8% complete data)*					
PROMIS Anxiety (T-Score)	52.4 ± 5.3	0.004	0.03	0.003	0.001
PROMIS Depression (T-Score)	50.1 ± 5.4	0.004	0.001	0.008	0.002
PCS (0–52)	13.3 ± 11.4	0.0001	0.004	0.0004	< 0.0001
SPCS walking (0–24)	3.0 ± 4.3	0.06	0.10	0.23	0.37
SPCS stair climbing (0–24)	2.5 ± 4.2	0.34	0.58	0.05	0.28
ESSI	26.2 ± 5.8	0.002	0.0007	0.002	0.002
Expectation for improvement (0–100)	88.0 ± 17.0	0.64	0.73	0.31	0.79
PANAS Positive affect	36.2 ± 7.3	0.18	0.13	0.88	0.81
BSI Somatization	1.7 ± 2.1	0.40	0.71	0.24	0.18
NEO Agreeableness	36.1 ± 5.7	0.008	0.02	0.01	0.02
NEO Extraversion	29.5 ± 6.8	0.02	0.02	0.02	0.04
NEO Neuroticism	14.2 ± 7.2	0.01	0.05	0.007	0.74
NEO Openness	27.9 ± 6.2	0.58	0.83	0.86	0.95
NEO Conscientiousness	36.2 ± 6.4	0.67	0.61	0.80	0.006
*Sleep-Related Factors (88.3% complete questionnaire data, 67.3% complete actigraphy data)*					
PSQI	8.1 ± 4.2	0.006	0.008	0.0002	0.001
ISI	9.6 ± 7.0	0.001	0.001	0.0002	0.002
Fatigue Severity Scale	30.5 ± 16.0	0.0004	< 0.0001	0.0003	< 0.0001
Sleep Efficiency (Actigraphy)	80.2% ± 9.5	0.09	0.35	0.34	0.12
WASO in minutes (Actigraphy)	54.5 ± 27.4	0.09	0.09	0.03	0.06
Total Sleep Time in minutes (Actigraphy)	389.9 ± 72.3	0.87	0.81	0.25	0.40
*QST-Assessed Factors (86.3% complete data)*					
Pain Sensitivity Questionnaire	4.9 ± 2.0	0.11	0.82	0.49	0.43
PPTh Trapezius (kPa)	411.1 ± 201.5	65	0.87	0.45	0.52
PPTh Patella (kPa)	532.9 ± 206.5	0.66	0.77	0.44	0.32
Cuff Algometry Pain Threshold (mmHg)	142.7 ± 51.7	0.04	0.19	0.07	0.06
Cold Pain Tolerance (sec)	75.0 ± 64.3	0.92	0.88	0.84	0.78
Cold Pain Aftersensations	20.3 ± 21.4	0.51	0.10	0.09	0.07
Conditioned Pain Modulation	123.8 ± 34.7	0.37	0.96	0.39	0.44
Temporal Summation (Patella)	13.2 ± 17.6	0.02	0.20	0.03	0.03
Temporal Summation (Finger)	10.8 ± 10.2	0.24	0.43	0.67	0.22

Psychosocially, study participants reported levels of distress consistent with general population norms, with T-scores for PROMIS measures of anxiety and depression quite close to 50. Catastrophizing scores on the PCS were similar to those observed in other samples of adults awaiting TKA [115, 116]. Neuroticism scores were low, social support was, on average, good, and mean expectations for improvement in pain following surgery were high (see Table 3). Sleep-related variables suggested significant levels of sleep disruption; mean ISI scores were in the range of “subthreshold insomnia”, and the majority of PSQI scores in the sample would place respondents into the category of “poor sleepers”; mean PSQI scores were very similar to past surveys of knee OA patients [117]. Actigraphy data suggested that, pre-operatively, participants were sleeping, on average, 6.5 h per night, with sleep efficiency of 80% and mild ratings of daytime fatigue (see Table 3).

Detail regarding surgical procedures was collected from the electronic medical record following discharge (see Table 3 for surgical variables). Surgeries generally lasted 1–2 h, and 37% of patients received general anesthesia. Slightly over 1/3 of participants received a femoral nerve or adductor canal block. On average, following their unilateral total knee replacements, participants in this study spent an average of 2.7 days in the hospital.

### TKA outcomes

Pre-operatively, patients reported moderate pain and functional impairment on the 4 outcome measures, BPI Pain Severity, BPI Pain Interference, WOMAC Pain, and WOMAC Physical Functioning (see Figs. 1–2). These measures were all strongly inter-correlated with one another, with correlation coefficients ranging from r = 0.70 (between BPI Pain Severity and Interference) to r = 0.80 (between WOMAC Pain and Physical Functioning), all *p*’s < 0.001. Repeated measures ANOVAs revealed that the mean values of all outcome measure improved significantly from pre-surgery to 6 months post-surgery on average (see Figs. 1 & 2, all *p*’s < 0.001); for example mean BPI Pain Severity and Interference scores dropped from nearly 4.5/10 prior to surgery to under 2/10 at 6 months after surgery. As expected, there was a good deal of variability in reports of pain and functional impact; for example, at the 6-month time point, while the majority of participants reported BPI Pain Severity scores under 2/10, a substantial minority (14.1%) reported pain severity of 4/10 or greater (see Table 2).

### Univariate associations

Table 3 shows univariate associations between pre-surgical predictors and 6-month outcomes. Interestingly, factors such as age and sex did not predict 6-month outcomes; education was the sole demographic factor that was associated with outcomes, with higher education predicting lower 6-month scores for WOMAC Pain and Function. Among clinical factors, the patient-reported presence of other chronic pain condition, a higher number of body areas in which pain was reported, and the pre-operative use of opioids was associated with higher BPI and WOMAC scores (i.e., more severe pain and functional impact of pain) following surgery. Surgical factors were essentially uncorrelated with 6-month outcomes. In the category of general health and functional factors, better patient-reported general health predicted better WOMAC outcomes. Pain intensity during the physical function tests (i.e., 6-min walk and stair-climbing), though not the functional performance on those tests, correlated with higher reported pain severity and pain impact at 6 months. Many of the measured psychosocial factors showed robust associations with pain and function outcomes, including negative affect-related measures such as anxiety, depression, neuroticism, and catastrophizing, which were consistently associated with more severe pain and pain-related dysfunction at 6 months after TKA. In addition, higher levels of social support and higher scores on the NEO subscales of agreeableness and extraversion were related to lower severity and impact of post-TKA pain. Patient-reported sleep measures showed a strong association with pain outcomes, though actigraphy-derived measures were less robustly associated. Higher scores on measures of sleep disruption (i.e., PSQI, ISI) and fatigue were correlated with more severe pain and functional impact. Finally, pre-surgical QST measures demonstrated modest and largely non-significant relationships with pain outcomes.

### Multivariable prediction models

The final multivariable model predicting BPI Pain Severity at 6 months after TKA had an *R*^2^ value of 0.34 (Table 4), with a number of significant and near-significant predictors. Higher pre-operative BPI scores, and higher reported levels of catastrophizing during the 6-min walk, were marginally associated with higher reported BPI Pain Severity at 6 months after TKA. Frankly significant (*p* < 0.05) risk factors included in the model were: higher catastrophizing, opioid use, the presence of another chronic pain condition, the number of painful body areas, and the degree of reported anxiety. Protective factors (i.e., predictive of lower Pain Severity) included higher sleep efficiency and higher agreeableness on the NEO.

**Table 4:** Final model for BPI Pain Severity at 6 months after surgery

**Pre-Operative Parameter**	**Estimate**	**95% Confidence Limits**		***P*-value**
BPI Pain Severity Pre-Surgery	0.12	-0.01	0.26	0.07
State PCS (Walking) Pre-Surgery	0.06	-0.009	0.13	0.09
PCS Total Pre-Surgery	0.02	0.002	0.05	0.04
Opioid Use Pre-Surgery	1.18	0.20	2.16	0.02
Sleep Efficiency Pre-Surgery	-0.03	-0.07	-0.007	0.02
Other Chronic Pain Pre-Surgery	0.78	0.18	1.39	0.01
Painful Areas Pre-Surgery	0.16	0.04	0.29	0.01
PROMIS Anxiety Pre-Surgery	0.07	0.02	0.12	0.006
NEO Agreeableness Pre-Surgery	-0.07	-0.12	-0.02	0.002

The final model has an R-square value of 0.34.

The final multivariable model predicting BPI Pain Interference at 6 months after TKA had an *R*^2^ value of 0.23 (the lowest explained variable of any of the 4 outcomes), with relatively few predictors selected for the final model (Table 5). Higher pre-operative BPI Pain Interference scores, along with higher levels of fatigue and depression, as well as patient report of experiencing neuropathic pain before surgery, were all marginally predictive of higher reported BPI Pain Interference at 6 months after TKA. The sole frankly significant (*p* < 0.05) predictor was social support; higher levels of support predicted lower Pain Interference after surgery.

**Table 5:** Final model for BPI Pain Interference at 6 months after surgery

Pre-Operative Parameter	Estimate	95% Confidence Limits		*P*-value
BPI Pain Interference Pre-Surgery	0.07	0.016	0.11	0.009
Fatigue Severity Pre-Surgery	0.008	-0.001	0.016	0.08
PROMIS Depression Pre-Surgery	0.02	-0.002	0.05	0.07
Neuropathic Pain Pre-Surgery	0.45	0.002	0.90	0.05
Social Support (ESSI) Pre-Surgery	-0.02	-0.05	-0.0002	0.04

The final model has an R-square of 0.23.

The multivariable model predicting WOMAC Pain at 6 months after TKA had an *R*^2^ value of 0.29 (Table 6). Pre-operative opioid use and more pain during the stair-climbing task before surgery were marginally predictive of higher reported WOMAC Pain at 6 months after TKA. Significant (*p* < 0.05) risk factors included in the model were: insomnia symptoms, higher levels of fatigue, and the number of painful body areas. Protective factors (i.e., predictive of lower Pain Severity) included higher educational attainment and higher agreeableness on the NEO.

**Table 6:** Final model for WOMAC Pain at 6 months after surgery

Parameter	Estimate	95% Confidence Limits		*P*-value
WOMAC Pain Pre-Surgery	0.04	-0.10	0.18	0.61
Opioid Use Pre-Surgery	0.99	-0.14	2.11	0.08
Stair-Climbing Pain Pre-Surgery	0.013	-0.001	0.03	0.06
ISI Total Pre-Surgery	0.45	0.02	0.88	0.04
Fatigue Severity Pre-Surgery	0.022	0.002	0.04	0.03
Education	-1.29	-2.41	-0.18	0.01
Painful Areas Pre-Surgery	1.59	0.40	2.77	0.009
NEO Agreeableness Pre-Surgery	-0.86	-1.30	-0.43	0.001

The final model has an R-square of 0.29.

Finally, the model predicting WOMAC Function (higher scores indicate more functional limitations) at 6 months after TKA had an overall *R*^2^ value of 0.31; higher pre-operative WOMAC Function scores as well as opioid use were marginally predictive of more reported WOMAC functional limitations at 6 months after TKA (Table 7). Significant (*p* < 0.05) risk factors included in the model were: increased pain during the 6-min walking task, as well as higher levels of general fatigue. Higher agreeableness on the NEO again emerged as a significant protective factor.

**Table 7:** Final model for WOMAC Function at 6 months after surgery

Parameter	Estimate	95% Confidence Limits		*P*-value
WOMAC Function Pre-Surgery	0.014	-0.001	0.03	0.06
Opioid Use Pre-Surgery	1.01	-0.10	2.12	0.07
Walking Pain Pre-Surgery	0.016	0.002	0.03	0.03
Fatigue Severity Pre-Surgery	0.03	0.014	0.05	0.0006
NEO Agreeableness Pre-Surgery	-0.09	-0.14	-0.04	0.0004

The final model has an R-square of 0.31.

### Moderating effects of QST

**Fig. 3:**
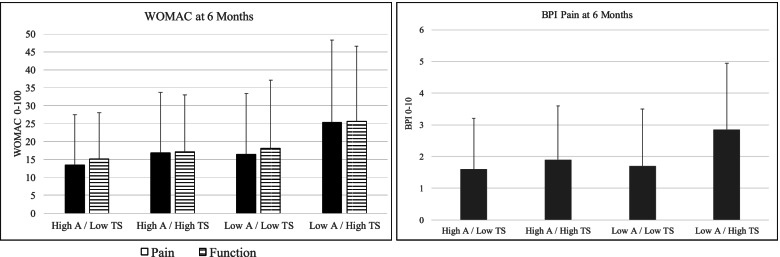
Interaction between Agreeableness and Temporal Summation on 6-month outcomes (means ± SD for subgroups). A = Agreeableness (measured with the NEO Inventory). TS = Temporal Summation of mechanical pain

Given the importance of QST-derived measures of pain responses in our earlier studies, we assessed the role of psychophysical variables as moderators of the observed relationships in this cohort. To explore these interactions, we identified the QST measure with the strongest relationship with each pain outcome, and then examined that factor as a potential moderator of significant psychosocial associations. In each case, we created simplified regression models examining the interaction between the most-strongly associated QST variable and psychosocial predictor. See Tables 8, 9, 10 and Fig. 3.

**Table 8:** Moderating effects of Temporal Summation on prediction of BPI Severity at 6 months

**Block**	**Variable**	**Beta**	**Block *R***^**2**^	***P*-value**
1	BPI Pain Severity	0.30	0.09	0.001
2	Agreeableness	-0.15	0.06	0.02
	PROMIS Anxiety	0.16		0.01
	Temporal Summation (Patella)	0.10		0.10
3	Agreeableness X Temporal Summation	-0.55	0.02	0.03
	Anxiety X Temporal Summation	0.15		0.23
Moderation Effects:				
**Low Temporal Summation**				
**Block**	**Variable**	**Beta**	**Block *R***^**2**^	***P*-value**
1	BPI Pain Severity	0.31	0.10	0.001
2	Agreeableness	-0.05	0.02	0.65
	PROMIS Anxiety	0.14		0.18
**High Temporal Summation**				
**Block**	**Variable**	**Beta**	**Block *R***^**2**^	***P*-value**
1	BPI Pain Severity	0.26	0.07	0.001
2	Agreeableness	-0.24	0.09	0.01
	PROMIS Anxiety	0.19		0.05

**Table 9:** Moderating effects of Temporal Summation on prediction of WOMAC Pain at 6 months

**Block**	**Variable**	**Beta**	**Block *R***^**2**^	***P*-value**
1	WOMAC Pain	0.25	0.07	< 0.001
2	Agreeableness	-0.24	0.09	< 0.001
	Painful Areas	0.20		0.002
	Temporal Summation (Patella)	0.07		0.22
3	Agreeableness X Temporal Summation	-0.49	0.03	0.02
	Painful Areas X Temporal Summation	0.25		0.06
Moderation Effects:				
**Low Temporal Summation**				
**Block**	**Variable**	**Beta**	**Block *R***^**2**^	***P*-value**
1	WOMAC Pain	0.38	0.13	< 0.001
2	Agreeableness	-0.15	0.02	0.11
	Painful Areas	0.04		0.73
**High Temporal Summation**				
**Block**	**Variable**	**Beta**	**Block *R***^**2**^	***P*-value**
1	WOMAC Pain	0.24	0.08	0.001
2	Agreeableness	-0.33	0.17	< 0.001
	Painful Areas	0.32		< 0.001

**Table 10:** Moderating effects of Temporal Summation on prediction of WOMAC Function at 6 months

**Block**	**Variable**	**Beta**	**Block *R***^**2**^	***P*-value**
1	WOMAC Function	0.37	0.13	< 0.001
2	Agreeableness	-0.19	0.06	< 0.001
	Fatigue Severity	0.15		0.002
	Temporal Summation (Patella)	0.05		0.22
3	Agreeableness X Temporal Summation	-0.58	0.02	0.05
	Fatigue Severity X Temporal Summation	0.07		0.51
Moderation Effects:				
**Low Temporal Summation**				
**Block**	**Variable**	**Beta**	**Block *R***^**2**^	***P*-value**
1	WOMAC Function	0.44	0.17	< 0.001
2	Agreeableness	-0.07	0.02	0.44
	Fatigue Severity	0.14		0.12
**High Temporal Summation**				
**Block**	**Variable**	**Beta**	**Block *R***^**2**^	***P*-value**
1	WOMAC Function	0.31	0.11	0.001
2	Agreeableness	-0.28	0.10	0.002
	Fatigue Severity	0.15		0.05

For BPI Pain severity, we observed a significant interaction of mechanical temporal summation on the patella with the predictive factor of agreeableness, such that the beneficial effect of agreeableness was most evident in the high temporal summation group. The subgroup who were low on agreeableness and high on temporal summation reported substantially higher 6-month pain severity than the other 3 subgroups (see Table 8 and Fig. 3). Similar patterns were evident for WOMAC Pain and Function outcomes: See Tables 8 and 9, and Fig. 3 for WOMAC outcomes. For both WOMAC Pain and WOMAC Function we observed a similar interaction, such that the effect of agreeableness was strongest in the high temporal summation group, with high agreeableness buffering the adverse impact of high temporal summation, and/or low agreeableness enhancing the detrimental impact of high temporal summation on post-surgical pain and functional outcomes. We did not test interactions for BPI Interference as no QST measures showed significant univariate prediction.

## Discussion

The present cohort study adds to a growing literature on the trajectory and determinants of pain-related outcomes following TKA. As in many prior reports and reviews [63, 118]), we found that a substantial minority (i.e., approximately 30%) of participants reported clinically significant ongoing pain and/or physical limitations 6 months after unilateral TKA. Reporting increased pain and physical dysfunction at 6 months post-TKA was predicted most consistently by higher levels of negative affect, prior pain history, and patient-reported sleep disruption. In addition, pain phenotypes such as more widespread pain and the presence of other chronic pain comorbidities were consistently associated with worse long-term outcomes. Surgical variables were not at all predictive of 6-month outcomes. Interestingly, lower levels of resilience-related “positive” psychosocial characteristics (i.e., lower agreeableness, lower social support) were among the strongest, most consistent predictors of poor outcomes. Maladaptive profiles of pain modulation, while not unique predictors, interacted with psychosocial risk factors such that the TKA patients with the most pain and dysfunction were those with lower resilience factors, combined with enhanced temporal summation. This study underlines the importance of considering psychosocial and psychophysical factors, as well as their interaction, in understanding postsurgical pain trajectories.

The growing number of joint replacement surgeries performed annually in the U.S. [27, 28] highlights the need to understand individual differences in post-operative pain and function in order to optimize surgical benefits and reduce the risk of poor outcomes. TKA outcomes show tremendous variability, with some patients reporting full resolution of knee pain and large improvements in function, while a significant minority of patients report continuing, or even worsening, pain and physical limitations [28, 118, 119]. Surgical patients who experience minimal benefit from surgery likely contribute importantly to the finding that a history of TKA is associated with reduced health-related quality of life for many years after the surgery itself [49, 52, 120]. Interestingly, reviews highlight the limited success of peri-operative interventions and pre-surgical exercise programs to improve long-term pain-related outcomes after knee replacement [121, 122]. It is certainly possible that the presence of multiple risk factors in a subset of patients limits the capacity of these treatments to substantially improve long-term outcomes, which further underscores the importance of identifying individuals whose pain is unlikely to improve after TKA.

Pre-surgical opioid use, though relatively uncommon in our sample (i.e., just under 10% of participants) was a significant univariate predictor of more severe pain and functional limitations at 6 months post-TKA, and emerged as a significant or near-significant factor in the multivariable analyses as well. This finding accords with prior studies of joint replacement [123], and other surgeries [124, 125]; in all cases, patients using opioids before surgery report more pain, greater analgesic requirements, and more complications after surgery. Pre-surgical opioid use has become more common in patients presenting for total joint replacement in the U.S., though recent reports suggest some reductions in the use of both pre- and post-surgical opioids [126, 127]. Such moves to limit opioid prescriptions and doses are consistent with CDC guidelines; based on a sizable literature documenting the harms of long-term and high-dose opioid usage, these trends should promote improvements in joint replacement outcomes.

To date, numerous studies, along with recent systematic reviews, report that psychosocial processes have important prospective influence in shaping the long-term course of post-TKA outcomes [36, 63]. The present results are consonant with that collective body of findings, in that anxiety, depression, and catastrophizing were robust univariable predictors of pain and functional outcomes, though because of their shared variance with each other and with related psychosocial factors, these measures appeared less consistently in multivariable models. In contrast, patient-reported measures of disrupted sleep and fatigue were strongly associated with more severe pain and more functional limitations at 6 months post-surgery on both the univariable and multivariable analyses, consistent with previous reports in the joint replacement literature [65, 128, 129]. Indeed, fatigue was among the most consistent multivariate predictors across outcomes at 6 months post-surgery. In multivariate models, actigraphy measures of sleep consolidation and self-reported insomnia severity, also contributed meaningful predictive explanatory variance in both 6-month BPI and WOMAC pain severity metrics, respectively. Because these sleep parameters have been found to be highly modifiable via cognitive-behavioral therapy for insomnia in knee OA patients [130, 131], these findings suggest a potential benefit of sleep interventions prior to surgery for post-surgical pain reduction.

One clearly understudied aspect of psychosocial functioning studied is the impact of positive, or resilience-related, psychosocial characteristics [67–69] on postsurgical outcomes after TKA. Overall, the present results suggest that “positive” factors such as agreeableness and social support (which show strong univariate associations with outcomes and which appear in the multivariable analyses as robustly significant predictors of pain and function at 6 months post-TKA) are critical contributors to long-term outcomes following joint replacement. Prior work has also highlighted the role of social support in facilitating improved outcomes after knee replacement [46, 132, 133]. Moreover, previous OA studies have illuminated the importance of social relationships in this population; for example, partner-supported interventions demonstrate enhanced benefits in OA patients [134–136]. Other non-pharmacologic treatments that enhance protective factors and reduce psychosocial risk factors (e.g., anxiety, catastrophizing) have shown promise in improving joint replacement outcomes as well [137].

As with psychosocial phenotyping, sensory profiling of knee OA patients in order to measure and quantify sensitization-related processes has become increasingly common. In cross-sectional comparisons, widespread hyperalgesia and higher scores on indices of central sensitization (e.g., temporal summation of pain) were observed in OA patients compared to controls [70, 72, 74]. In the present study, some pre-surgical QST variables were predictive of 6-month outcomes (e.g., higher temporal summation was associated with more pain and physical dysfunction), though the strength of these relationships was modest. Sensory factors were perhaps most influential in their interactive relationship with some psychosocial factors. The patients with the most severe pain and physical limitations at 6 months after TKA were those with both high levels of temporal summation and low levels of agreeableness pre-surgically. Interestingly, several prior studies have also reported synergistic interactions among risk factors. For example, among patients undergoing total joint arthroplasty, preoperative depression predicted poorer outcomes (e.g., more severe pain) after surgery, but only in patients using opioids pre-surgically [138]. Similarly, among patients undergoing breast surgery, pain-modulatory factors assessed with QST and psychosocial factors such as catastrophizing interacted with the presence of chronic pain to predict post-operative outcomes [139]. While prior work has evaluated synergistic effects of multiple risk factors, the present results may be among the first to combine resilience factors (e.g., agreeableness) with risk factors (e.g., high temporal summation of pain) to predict long-term outcomes after TKA.

A number of study limitations should temper interpretation of these findings. First, though we recruited participants from multiple sites, the sample is largely white and likely does not fully reflect the U.S.’s full demographic diversity. Second, the outcome measures are entirely self-report, which is typical in pain outcome studies, but some investigations of post-operative outcomes do include provider-based measures as well, which would be of interest in future studies. Third, while 6 months after surgery is a common primary assessment point, some TKA outcome studies follow participants for years after their surgeries, providing truly long-term outcome data. It is certainly possible that some of the current study subjects, who reported substantial pain and disability at 6 months, went on to make full recoveries in the months thereafter. Finally, our sample size does not permit full modeling of all possible interactions between risk or resilience factors, and these findings should accordingly be considered exploratory. Therefore, larger sample sizes will be necessary to fully model the interplay between all of these predictive variables.

## Conclusions

Collectively, these findings highlight the complex multidimensional nature of pain, the variability in trajectories of post-TKA pain, and the potential contributory role of psychosocial, sleep-related, and pain-modulatory factors in shaping these outcomes. Globally, an improvement in the precision of our predictive models will eventually help to develop and deliver targeted, personalized treatments that can reduce the incidence and impact of persistent post-operative pain [31, 34, 78]. The present findings may be particularly helpful in identifying which predictive domains should be included in the development of risk algorithms. Based on the present findings, measures of negative affect and psychosocial distress, the presence of other chronic pain conditions, especially widespread pain, sleep disruption, opioid use, education, social support, and an interpersonal measure such as agreeableness should be considered for inclusion in these models. In addition, considerations of synergy or interactions among risk factors may be important, as the present findings suggest.

## Data Availability

The datasets used and analyzed during the current study are available from the corresponding author on reasonable request.

## Abbreviations

BMI: Body Mass Index
BPI: Brief Pain Inventory
CPM: Conditioned Pain Modulation
CPT: Cold Pressor Task
ISI: Insomnia Severity Index
OA: Osteoarthritis
PCS: Pain Catastrophizing Scale
PPTh: Pressure Pain Threshold
PROMIS: Patient Reported Outcome Measurement Information System
PSQ: Pain Sensitivity Questionnaire
PSQI: Pittsburgh Sleep Quality Index
QST: Quantitative Sensory Testing
TKA: Total Knee Arthroplasty
TKR: Total Knee Replacement
WOMAC: Western Ontario McMaster Universities Scales
WPI: Widespread Pain Inventory
